# Comparative Analyses of *Lycodon rufozonatus* and *Lycodon rosozonatus* Gut Microbiota in Different Regions

**DOI:** 10.1002/ece3.70480

**Published:** 2024-10-22

**Authors:** Fei Zhu, Ke Sun, He Zhang, Jing Lu, Peng Guo, Jiaqi Zhang, Yu Xu, Bing Lyu

**Affiliations:** ^1^ School of Life Sciences Guizhou Normal University Guiyang Guizhou China; ^2^ Guizhou Academy of Forestry Guiyang Guizhou China; ^3^ Faculty of Agriculture, Forestry and Food Engineering Yibin University Yibin Sichuan China

**Keywords:** different regions, gut microbiome differences, *Lycodon rosozonatus*, *Lycodon rufozonatus*, microbial diversity

## Abstract

The interactions between hosts and the gut microbiota are intricate and can significantly affect the ecology and evolution of both parties. Various host traits, including taxonomy, diet, social behaviour, and external factors such as prey availability and the local environment, all play an important role in shaping composition and diversity of the gut microbiogta. In this study, we explored the impact of intestinal microorganisms on the host in adapting to their respective ecological niches in two species of snakes. We collected feces from *Lycodon rufozonatus* and *Lycodon rosozonatus* from different geographical locations and used 16S rRNA gene sequencing technology to sequence the v3‐v4 region. The results revealed that there was no significant difference in the alpha diversity of intestinal microorganisms between *L. rufozonatus* and *L. rosozonatus*. The gut microbiota of all individuals comprised four main phyla: Pseudomonadota, Bacteroidota, Bacillota, and Actinomycetota. At the genus level, the genus *Salmonella* dominated the enterobacterial microbiota in the samples from Hainan, while there was no obvious dominant genus in the enterobacterial microbiota of the samples from the other four localities. Comparative analysis of enzyme families annotated to the gut microbiota between *L. rufozonatus* and *L. rosozonatus* from the four sampling regions by CAZy carbohydrate annotation revealed that nine enzyme families differed significantly in terms of glycoside hydrolases (GHs). In addition, we compared the composition of gut microbial communities between *L. rufozonatus* and *L. rosozonatus* and investigated the impact of the differences on their functions. Our results will provide insights into the coevolution of host and gut microbes.

## Graphical abstract

This study delved into the intricate relationship between hosts and their gut microbiota, highlighting how various factors such as taxonomy, diet, and environmental conditions influence microbial composition. Focusing on two snake species, *Lycodon rufozonatus* and *Lycodon rosozonatus*, from different locations, fecal samples were analyzed using 16S rRNA gene sequencing.

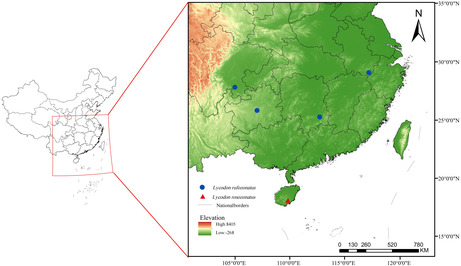

## 1 Introduction

A wide variety of microorganisms live in the gastrointestinal tract of all animals. These microorganisms have a significant impact on host biology and host fitness (Cryan and Dinan 2012; Thaiss et al. 2016). Interactions between host and microbiome affect metabolism and contribute to host adaptation to changing environments (Wang, Shang et al. 2022; Zhu, Qi, et al. 2022; Zhu, Wang, and Bahrndorff 2021). Similarly, the host diet affects the physiology and morphology of many taxa (Karasov and Martínez del Rio 2007), affecting the composition of the gut microbiota (Ley and Hamady 2008; McFall‐Ngai et al. 2013; Moran, Ochman, and Hammer 2019). In addition to dietary and genetic variation, geographical environment and physicochemical characteristics also affect the composition of the intestinal microbiota (Bestion et al. 2017; Indest et al. 2018; Trevelline et al. 2019); furthermore, the gut microbes and their respective animal hosts have coevolved over a long period of time to achieve balance and reciprocity (Ji et al. 2014; Wang, Wu et al. 2022; Zhu et al. 2022).

The coexistence of the host and its gut microbiota depends on the influence of several different factors, such as host genotype, gut morphology, physiological state, the immune system, and environmental perturbations (Aivelo and Norberg 2018; Bestion et al. 2017; Moeller et al. 2020). Despite increasing research, the rate at which gut microbiota can change and the relative impact of diet compared to other environmental factors in driving these changes remain poorly understood (Lemieux‐Labonté et al. 2022).

Recently, research on gut microbiota has concentrated on the differences in gut flora among species across varying altitude gradients, habitat environments, and dietary sources (Becker, Hill, and Butaye 2021; Jiang et al. 2017; Wu et al. 2020; Xu et al. 2020; Zhang et al. 2016). For instance, gut microbes of small indian mongoose (*Urva auropunctata*) in the Caribbean show significant variation between environments, reinforcing the concept that gut microbial composition reflects feeding ecology and host evolutionary history (Becker, Hill, and Butaye 2021); in a study of the diet of crocodile lizard (*Shinisaurus crocodilurus*), it was observed that changes in diet influence variations in the composition of intestinal microorganisms and are closely associated with the occurrence of diseases (Jiang et al. 2017). Most studies revealed a coevolutionary relationship between gut microbes and their hosts, and the gut microbes assisted the hosts in adapting to different environments.

The gut microbiota of vertebrates is known to be highly plastic and able to change in response to environmental changes to help the animal adapt to new condition (Alberdi et al. 2016). For example, the diet and habitat of the eastern water dragon (*Intellagama lesueurii*) had an effect on its gut microbiome, with lizards living in urban areas exhibiting greater bacterial diversity than populations living in natural habitats (Littleford‐Colquhoun et al. 2019). Other habitat characteristics also influence the lizard microbiome. For example, changes in temperature led to changes in the composition of intestinal microorganisms in the common lizard (*Zootoca vivipara*), increased temperature also led to a reduction in microbial diversity and an increase in pathogenic bacteria (Bestion et al. 2017). During the ex situ conservation of forest musk deer (*Moschus berezovskii*), the diversity of its intestinal microbial flora was significantly reduced, indicating that its microbial diversity has been altered by habitat changes (Jiang et al. 2022). Some studies have found that gut bacteria may vary between closely related species, potentially providing distinct adaptations and enabling differentiation in niches and habitats. For example, in the study of the intestinal microorganisms of two sea snakes by Zhong et al. (2022) and two species of fox gut microbes by Wang, Shang et al. (2022), both studies demonstrated differences in the gut microbiota between the two species. Study of *loggerhead turtles* from the United States and Australia revealed that their intestinal microbial composition in two regions was significantly different, suggesting that the microbiota of *loggerhead turtles* is strongly affected by geography (Scheelings et al. 2020). Tropical forests can provide hosts with a wider range of food sources than simpler environments (Jácome‐Hernández et al. 2023), and the diversity of the gut microbiota is also greater. Furthermore, social interactions between hosts can also influence the gut microbiome in many animal species, although these mechanisms remain less studied (Archie and Tung 2015).

An increase in host intestinal microbial diversity is positively correlated with enhanced metabolic activity and energy acquisition (Ley, Lozupone et al. 2008). Conversely, a reduction in microbial diversity or an increase in harmful taxa can lead to metabolic disorders and inflammatory responses in the host (Cotillard et al. 2013). The community structure and relative abundance of intestinal microorganisms differ among hosts (Zhang et al. 2019; Shang et al. 2023).

Reptiles have a wide variety of life history traits, particularly in terms of diet and reproductive patterns (Shine and Bonnet 2000; Vitt and Caldwell 2013). Studies on intestinal microorganisms have proven that the intestinal microbiota can affect or even regulate host metabolism (Li et al. 2008), nutrition (Kohl, Stengel, and Dearing 2016), immunology (Hooper, Littman, and Macpherson 2012), behavior (Ezenwa et al. 2012), morphology (Broderick, Buchon, and Lemaitre 2014) and development (Gilbert, Bosch, and Ledón‐Rettig 2015). Hoffbeck et al. (2023) sequenced gut microbes from 91 reptile species using 16S rRNA genes and found significant differences in alpha and beta diversity between host order, environment, diet, habitat and conservation status, with host diet and order having the greatest influence on these differences. Notably, differences in food resources are the most direct and important factors influencing the diversity and abundance of the gut microbiota (Yun et al. 2014; Zhou et al. 2020).

The snakes are very important group of vertebrates. However, research into snakes gut microbiology were few and scattered (Hoffbeck et al. 2023). *Lycodon rufozonatus* and *Lycodon rosozonatus* are two closely related congeners of snake family Colubridae (Wang et al. 2020). *Lycodon rufozonatus* is mainly distributed in China, Japan, South Korea, Laos, Vietnam, Russia and other regions, while, *L. rosozonatus* was endemic to Hainan Province, China. In this paper, we studied and analyzed the gut microbiota composition of two closely related species from different geographical locations to speculate on the adaptive evolution of the gut microflora of *L. rufozonatus* and *L. rosozonatus* in association with their hosts.

## 2 Materials And Methods

### 2.1 Sample Collection

**FIGURE 1:**
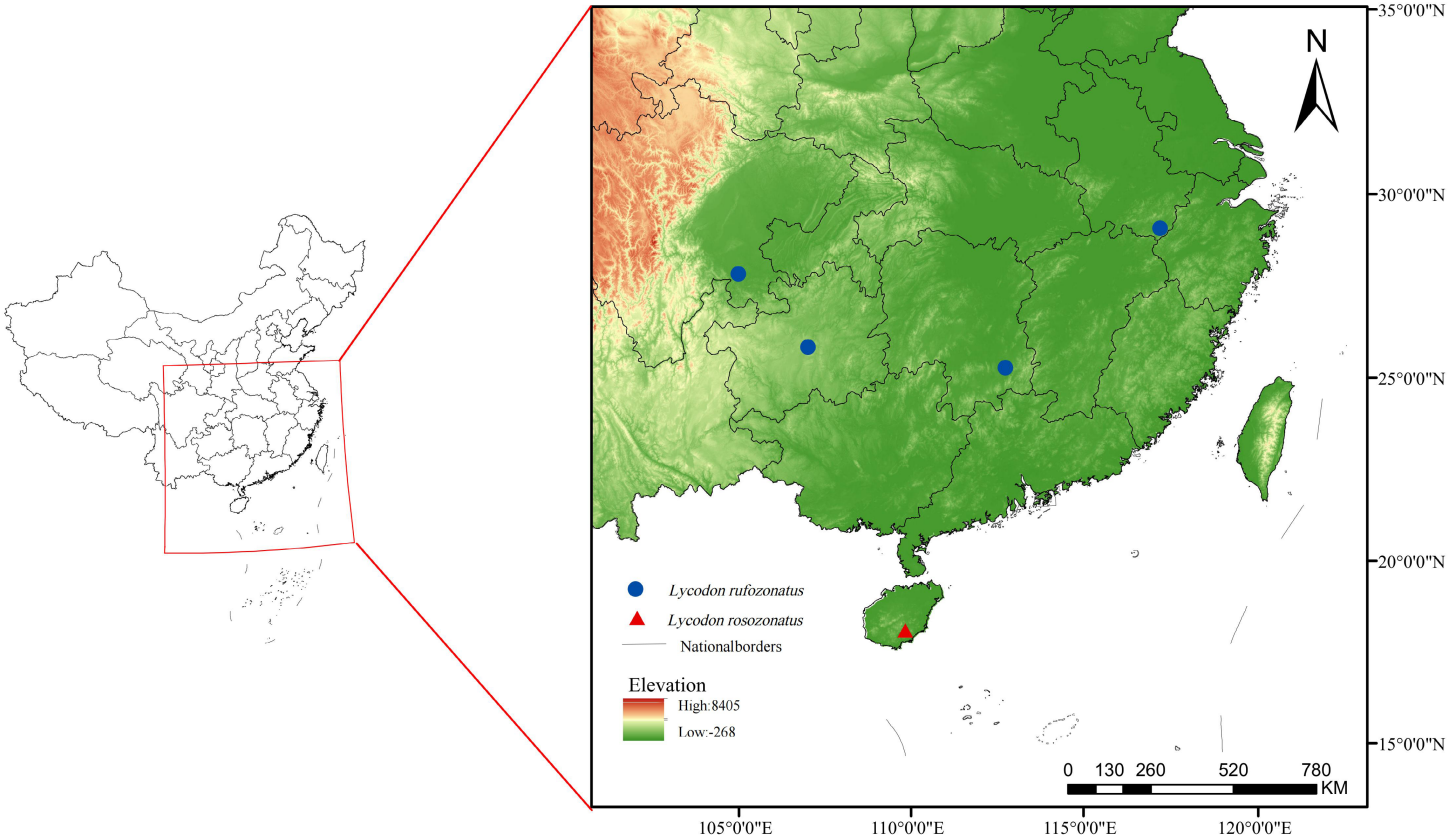
Map showing sample localities.

In total, 21 specimens of the two species were collected from different regions (Figure 1). Of them, three individuals of *L. rosozonatus* (GS0676, GS0689, and GS0690) were collected at the Diaoluoshan National Nature Reserve in Hainan Province (109.88137 E, 18.672096 N; 451 m) in 2023; 18 individuals of *L. rufozonatus* from Guiyang city, Guizhou Province (GS0691, GS0692, GP9686, GP10137, and GP10138. 106.898242 E, 26.784723 N; 1132 m), Yibin city, Sichuan (GP10817, GP10819, and GP10835. 104.657396 E, 28.812890 N; 517 m), Yongxing County, Hunan Province (GP10161, GP10829, GP10142, GP10143, and GP10144. 113.12772274 E, 26.08781426 N; 161 m), and Huangshan city, Anhui Province (GP9292, GP9218, GP12533, GP12570, and GP12571. 118.31657410 E, 29.73509168 N; 180 m). The specimens were preserved at the Yibin Key Laboratory of Animal Diversity and Ecological Conservation at Yibin University (GP series) and the Animal Diversity and Evolution Laboratory at Guizhou Normal University (Table S1).

### 2.2 DNA Extraction and Sequencing

Under aseptic conditions, we collected, squeezed, and scraped the entire intestine of all samples to obtain sufficient contents and transferred them to sterile centrifuge tubes containing 95% alcohol. Before DNA extraction, the sterile centrifuge tubes were frozen at −80°C to preserve the samples. Genomic DNA was extracted from the gut contents by a commercial company (Novogene, Beijing, China) utilizing a DNA extraction kit (Catalog number: DP336‐T3) following the provided instructions. We amplified the V3‐V4 region of the bacterial 16S rRNA gene.

The genomic DNA was randomly sheared into short fragments. The obtained fragments were end repaired, A‐tailed and further ligated with an Illumina adapter. The fragments with adapters were PCR amplified, size selected, and purified. The library was checked with a Qubit fluorometer and real‐time PCR for quantification and a bioanalyzer for size distribution detection. The quantified libraries were pooled and sequenced on Illumina platforms according to the effective library concentration and the amount of data needed.

### 2.3 Data Quality Control

Readfq (V8, https://github.com/cjfields/readfq) was used for converting the raw fluorescence image files acquired from the Illumina platform into short reads (raw data) through base calling. These short reads were then documented in FASTQ format, which contained sequence information and corresponding sequencing quality details.

Sequence artifacts, such as reads with adapter contamination, low‐quality nucleotides, and unidentifiable nucleotides (N), pose challenges to reliable bioinformatics analysis. Hence, quality control is a crucial step to ensure meaningful downstream analyses. We utilized Fastp (version 0.19.7) for basic statistics on raw read quality. The data processing included the following steps: (1) discarding paired reads containing adapter contamination; (2) discarding paired reads if more than 10% of bases were uncertain in any read; and (3) discarding paired reads if the proportion of low‐quality (Phred quality < 5) bases exceeded 50% in any reads.

### 2.4 Statistical Analysis

The host database was queried using Bowtie software (Bowtie 2.2.4) to filter out host‐derived reads and identify clean ones (Langmead and Salzberg 2012; Langmead et al. 2019). After host removal, the sequences were aligned to standard archaea, bacteria, human, UniVec_Core, and viral databases using Kraken software (Kraken 2.0.7) to obtain annotation information and abundance tables, swiftly classifying sequencing reads into species (Karlsson et al. 2012, 2013).

We determined the relative abundance of major taxa by filtering the total relative abundance of operational taxonomic units (OTUs) to 0.1% and expressed the data as averages. Rarefaction curves were constructed to assess sequencing depth using the vegan, BiodiversityR, and ggplot2 packages in R (R 4.2.2 package), and the alpha diversity matrix was generated based on the observed OTUs, encompassing the observed Sob species, Chao1, Shannon diversity index, and Simpson index derived from the relative abundance of the observed OTUs. Differences between the two species were examined using the Wilcoxon t test. To assess changes in the intestinal microbial structure between samples of the two species, we utilized R's vegan and ggplot2 software packages to conduct PCoA (principal coordinate analysis) and NMDS (nonmetric multidimensional scaling) based on the Bray–Curtis distance matrix. The ComplexHeatmap package in R facilitated clustering heatmap creation and analysis. Analysis of similarity based on the Bray–Curtis distance metric (ANOSIM) was employed to evaluate *L. rufozonatus* in the Guizhou, Sichuan, Hunan, and Anhui sampling areas and *L. rosozonatus* in the Hainan sampling area. Additionally, the online LEfSe tool was employed to generate LDA histograms and cladograms, while linear discriminant analysis (Segata et al. 2011) was used to identify dominant OTUs in the two species to pinpoint differences between distinct sampling areas.

### 2.5 Functional Analyses

Cd‐hit (Fu et al. 2012) software was used to cluster and eliminate redundancy in the sequences assembled by Megahit (Li et al. 2015), resulting in a clustered sequence list and a nonredundant sequence list. For quantitative analysis, host‐depleted Fastq sequences and gene sequences annotated by Prodigal (Hyatt et al. 2010) for proteins were used for Salmon (Patro et al. 2017) analysis to obtain quantitative data for each sample, which were then summarized using Excel. The nonredundant sequences were annotated into protein sequences via Prodigal software, a run_dbcan (Zhang et al. 2018) database was established, and the CAZy carbohydrate enzyme database (version 2018‐01, http://www.cazy.org/) (Cantarel et al. 2009) was utilized for annotation to acquire corresponding information. The Complex Heatmap package in R was used to construct and analyze the cluster heatmaps. Using STAMP software, we identified enzyme families with significant differences.

## 3 Results

### 3.1 Sequencing Data

**FIGURE 2:**
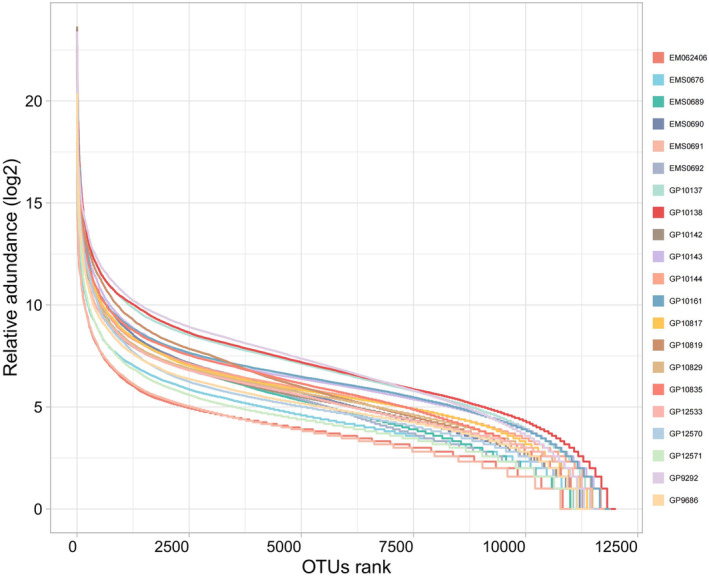
Runk‐abundance curve of the gut microbiota of *Lycodon rufozonatus* from Guizhou, Sichuan, Hunan and Anhui; and *Lycodon rosozonatus* from the Hainan. Each curve represents one sample and is labeled with a different color.

In this study, we collected 21 samples, consisting of 3 *L. rosozonatus* and 18 *L. rufozonatus*. Sequencing was conducted using the Illumina PE350 sequencing platform, yielding a total of 632,778,554 raw reads, with sample reads ranging from 47,019,574 to 179,209,80. After quality filtering, 622,584,924 clean reads were obtained, each ranging from 45,905,122 to 17,813,950 (Table 1). According to the 95% similarity level test, a total of 12,612 OTUs were acquired, with an effectiveness rate exceeding 95%. The average sequence length was 350 bp, covering the full extent of the V3–V4 region and adhering to random statistical standards. Upon the leveling off of the rank‐abundance curve (Figure 2), a satisfactory number of OTUs were identified at this sequencing depth and generally stabilized; this indicates their ability to accurately portray the microbial community, and the results are adequate for estimating microbial diversity.

**TABLE 1:** Statistics of the sequencing data preprocessing and quality control.

Location	Sample	Raw reads	Clean reads	Effective (%)
Hainan	EMS0676	47019574	45905122	97.63
Hainan	EMS0689	34783500	34458832	99.07
Hainan	EMS0690	35998102	35590320	98.87
Guizhou	EMS0691	35189910	34659052	98.49
Guizhou	EMS0692	35336649	35012898	99.08
Guizhou	GP10137	19801005	19786550	99.92
Guizhou	GP10138	18975810	18957015	99.90
Guizhou	GP9686	18484095	18316410	99.09
Hunan	GP10142	18592105	18581010	99.94
Hunan	GP10143	18703370	18687060	99.91
Hunan	GP10144	17920980	17813950	99.40
Hunan	GP10161	33581676	33295870	99.15
Hunan	GP10829	34178214	32625756	95.46
Sichuan	GP10817	33211482	32595218	98.14
Sichuan	GP10819	34556178	34025592	98.46
Sichuan	GP10835	25741332	24863108	96.59
Anhui	GP12533	39407602	38692442	98.19
Anhui	GP12570	35047674	34180232	97.52
Anhui	GP12571	38195230	37326316	97.73
Anhui	GP9292	21370160	21348705	99.89
Anhui	EM062406	36683906	35863466	97.76

### 3.2 The Composition and Structure of Intestinal Bacteria

**FIGURE 3:**
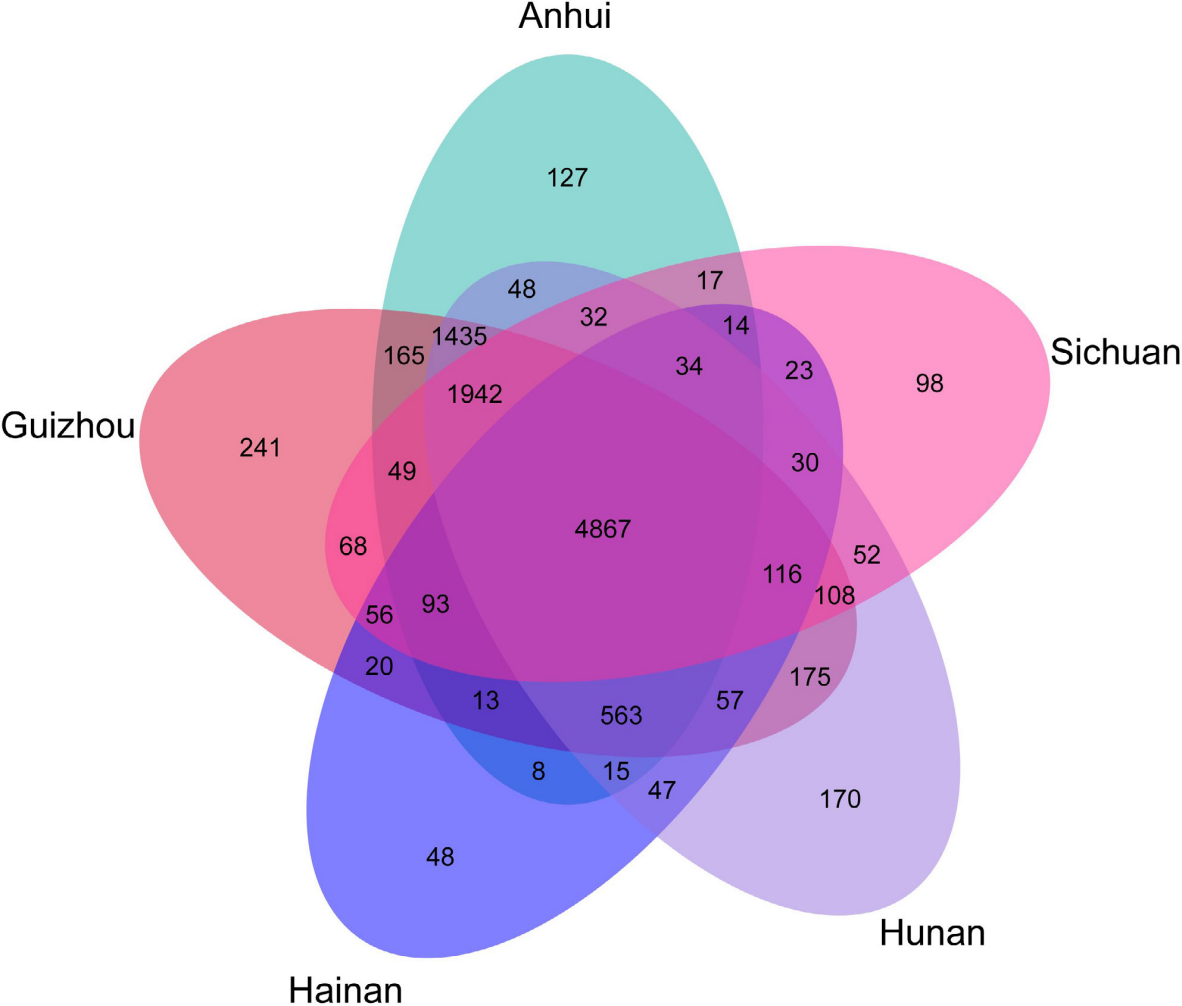
Venn diagrams of the gut microbiota of *Lycodon rufozonatus* from Guizhou, Sichuan, Hunan and Anhui; and of *Lycodon rosozonatus* from Hainan. The five different colored ovals in the figure represent five sampling areas, and the letters on each ellipse correspond to the corresponding sampling area. The numbers in the figure represent the number of OTUs unique or common to each plot.

In this study, a classification study was conducted on the gut microbiota of *L. rufozonatus* from four localities, Guizhou, Sichuan, Hunan and Anhui, and *L. rosozonatus* from Hainan. We identified bacterial OTUs from a total of 47 phyla, 101 classes, 214 orders, 515 families, 1957 genera, and 9777 species. Based on the Venn diagram, the gut microbiota of *L. rufozonatus* from four localites and *L. rosozonatus* from Hainan that 4867 OTUs were shared by all samples; there were unique gut microbiota OTUs in each sampling area. Among them, there were 48 in Hainan, 241 in Guizhou, 170 in Hunan, 98 in Sichuan and 127 in Anhui (Figure 3).

**FIGURE 4:**
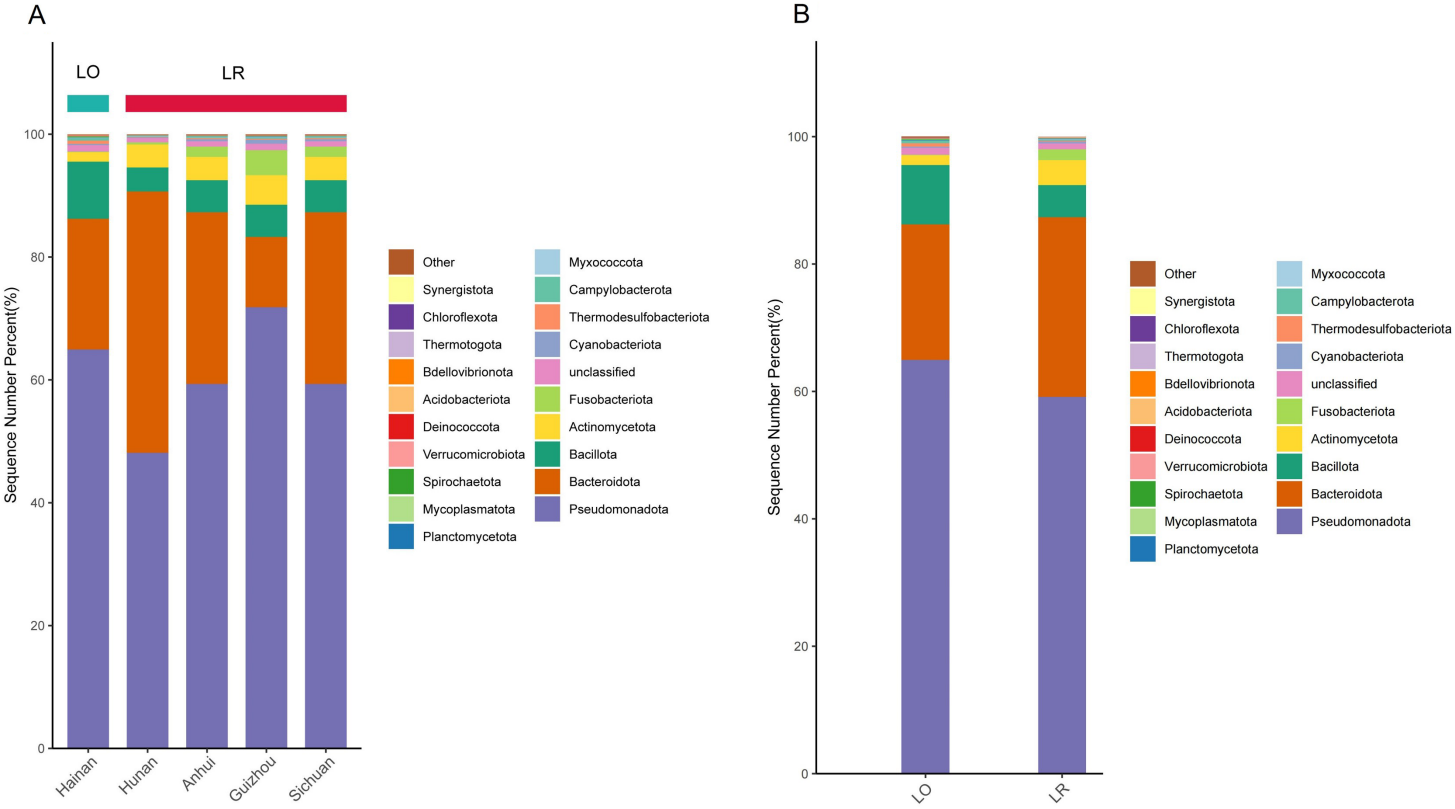
(A) The relative abundance of the top 20 phyla in the gut microbiota of *Lycodon rufozonatus* from Guizhou, Sichuan, Hunan and Anhui, and *Lycodon rosozonatus* from Hainan. (B) Relative abundance of *Lycodon rufozonatus* and *Lycodon rosozonatus* at the level of the top twenty phyla of the gut microbiota.

**FIGURE 5:**
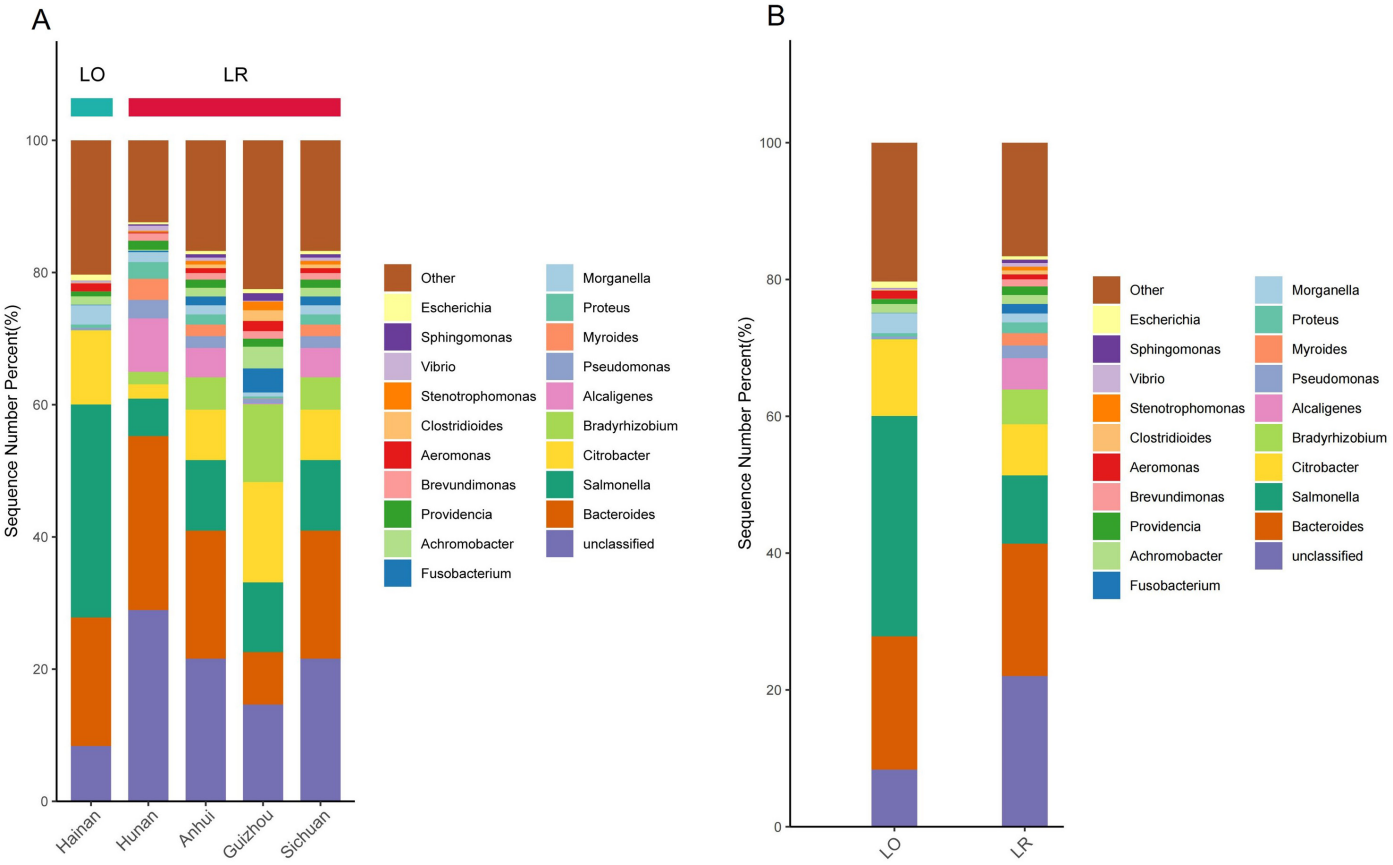
(A) Relative abundance of the top twenty genus‐level gut microbiota of *Lycodon rufozonatus* from Guizhou, Sichuan, Hunan and Anhui, and *Lycodon rosozonatus* from Hainan. (B) The relative abundance of the top twenty phyla and genera in the gut microbiota of *Lycodon rufozonatus* and *Lycodon rosozonatus*.

At the phylum level, the top four bacterial phyla of the intestinal microbiota of *L. rufozonatus* from Guizhou, Sichuan, Hunan and Anhui and of *L. rosozonatus* from Hainan were Pseudomonadota (59.56 ± 19.95%), Bacteroidota (21.67 ± 14.34%), Bacillota (8.87 ± 5.52%), and Actinomycetota (4.39 ± 3.25%), respectively (Figure 4A,B). At the genus level, the five bacterial genera with the greatest relative abundances in the Guizhou sampling area were *Citrobacter* (15.18 ± 14.80%), unclassified (14.63 ± 9.48%), *Bradyrhizobium* (11.76 ± 11.12%), *Salmonella (*10.55 ± 5.81%), and *Bacteroides* (7.94 ± 7.73%). The five bacterial genera with the greatest relative abundances in the Sichuan sampling area were unclassified (15.37 ± 9.4%), *Morganella* (14.88 ± 17.935), *Bacteroides* (12.56 ± 8.16%), *Citrobacter* (12.40 ± 13.49%), and *Vibrio* (9.79 ± 7.53%). The five bacterial genera with the five genera with the greatest relative abundances in the Hunan sampling area were unclassified (28.92 ± 14.73%), *Bacteroides* (26.32 ± 13.81%), *Alcaligenes* (8.10 ± 17.86%), *Salmonella* (5.67 ± 8.24%), and *Myroides* (3.19 ± 5.10%). The five bacterial genera with the greatest relative abundances in the Anhui sampling area were *Bradyrhizobium* (29.42 ± 24.43%), *Achromobacter* (15.47 ± 22.83%), unclassified (6.77 ± 12.96%), *Providencia* (3.97 ± 5.57%), and *Bacteroides* (2.99 ± 11.17%). The above data show that among the five bacterial genera with relative abundances at the genus level, Citrobacter, *Bradyrhizobium*, *Salmonella*, and *Bacteroides* were the most abundant in the four sampling areas of Guizhou, Sichuan, Hunan, and Anhui. Among *L. rosozonatus* in the Hainan sampling area, the five most abundant bacterial genera were mainly *Salmonella* (32.23 ± 19.69%), *Bacteroides* (19.45 ± 13.27%), *Citrobacter* (11.18 ± 6.37%), and *Morganella* (2.92 ± 1.67%) (Figure 5A,B).

### 3.3 Gut Microbial Diversity

**FIGURE 6:**
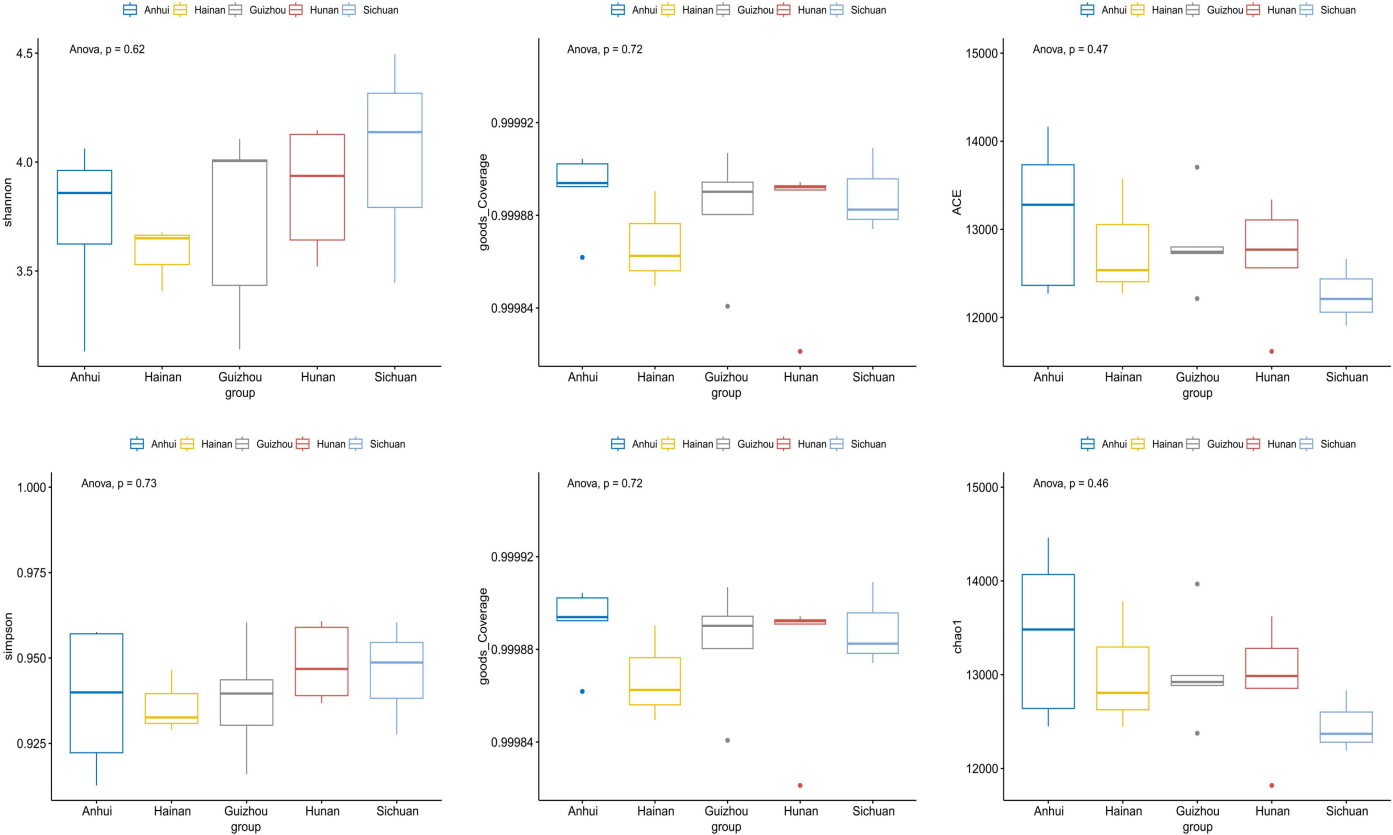
ANOVA test on alpha diversity of gut microbiota of *Lycodon rufozonatus* from Guizhou, Sichuan, Hunan, and Anhui; and *Lycodon rosozonatus* from Hainan.

We calculated the alpha diversity indices of the different samples (Table S2), and the Goods_Coverage indices all exceeded 99.8%, indicating that sufficient 16S rRNA gene sequences were obtained from the intestines of *L. rufozonatus* in the Guizhou, Sichuan, Hunan, and Anhui sampling areas and from *L. rosozonatus* in the Hainan sampling area. According to the ANOVA results between the alpha diversity of *L. rufozonatus* in the four sampling areas and the alpha diversity of *L. rosozonatus* in the Hainan sampling area, there was no significant difference between them (Figure 6) (*p* > 0.05).

**FIGURE 7:**
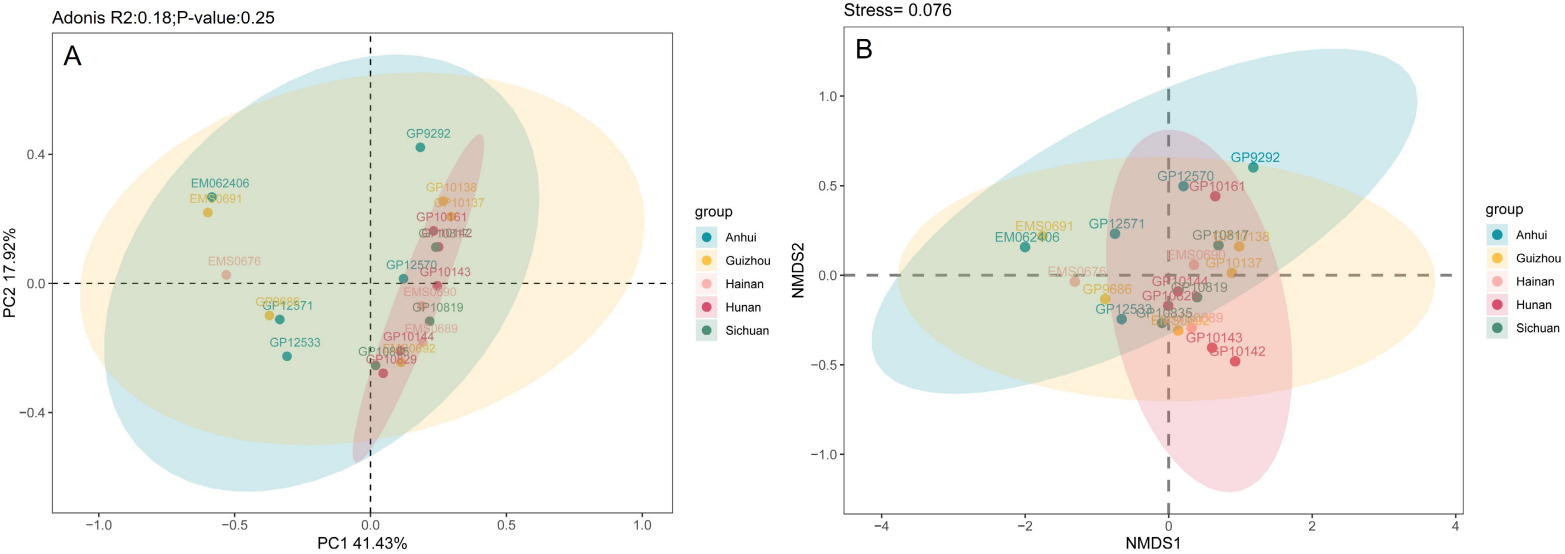
(A) Distance analysis of the gut microbiota of *Lycodon rufozonatus* in Guizhou, Sichuan, Hunan, and Anhui sampling areas and *Lycodon rosozonatus* in Hainan based on PCoA. Each point represents a sample, and the closer the distance between sample points, the higher the similarity. (B) Distance analysis of the gut microbiota of *Lycodon rufozonatus* and *Lycodon rosozonatus* in the sampling areas of Guizhou, Sichuan, Hunan, and Anhui based on NMDS. Each point represents a sample, and the distance between points represents the degree of difference.

Beta diversity was assessed by PCoA and NMDS analysis of the gut microbiota. According to the PCoA (principal coordinate analysis) results, the sample localities of *L. rufozonatus* and *L. rosozonatus* were relatively evenly distributed. Two sample localities overlap in the sampling interval, and all samples overlap. There is no obvious difference in the distance between sample points in the area (Figure 7A,B). Adonis analysis indicated a low R value (*R* = 0.2, *p* = 0.452), which is close to 0, suggesting that there was no significant difference between or within the groups. According to the results of NMDS (nonmetric multidimensional scaling) analyses, the distributions of *L. rufozonatus* in the four sampling areas and of *L. rosozonatus* in the Hainan sampling area were relatively consistent, with some overlapping results between sampling areas and no aggregation of samples within all sampling areas.

### 3.4 Differences in the Gut Microbiota Between *L. rufozonatus* and *L. rosozonatus*

**FIGURE 8:**
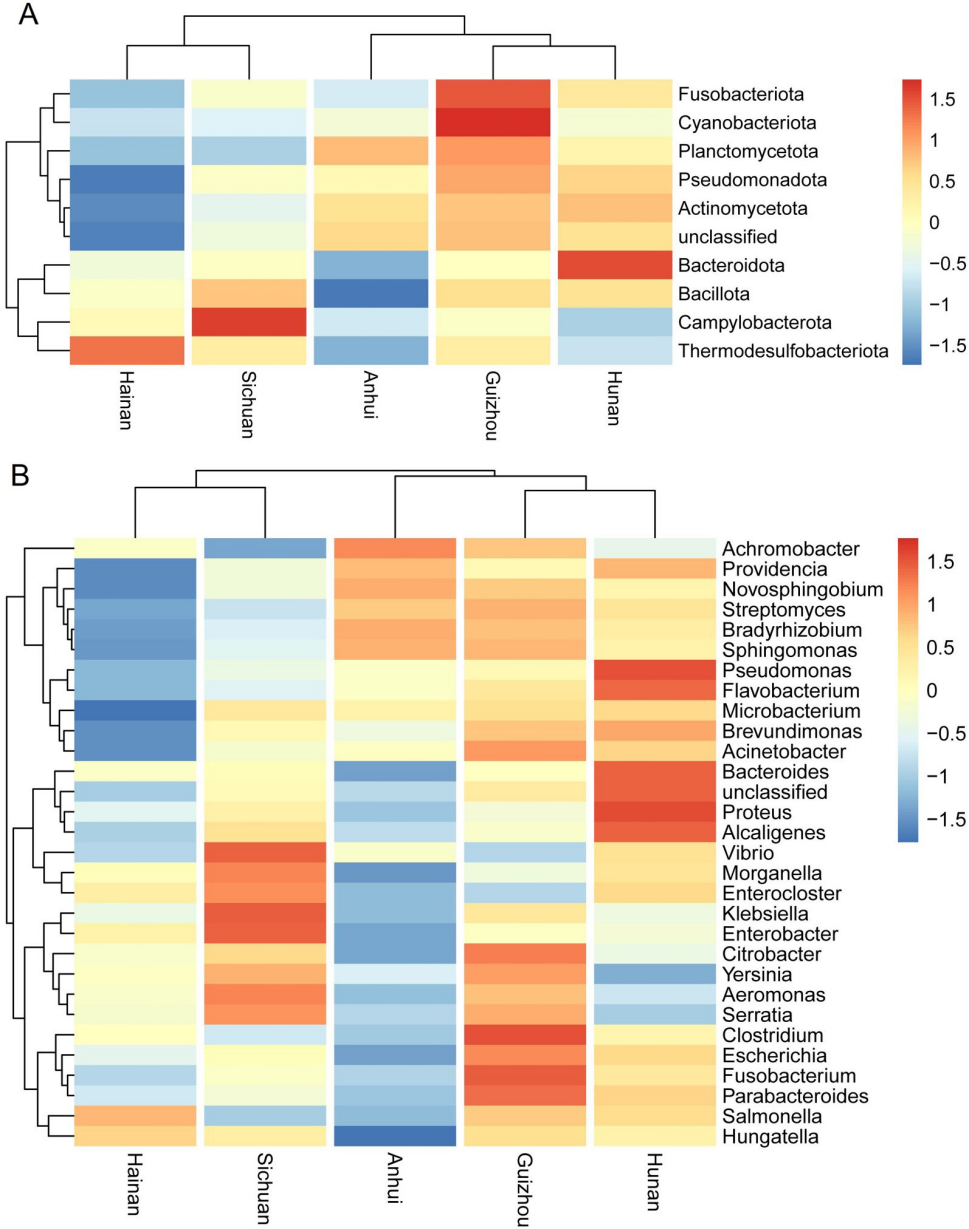
(A) Results of the top 10 phylum‐level clustering heatmap of relative abundance between the gut microbiota of *Lycodon rufozonatus* and *Lycodon rosozonatus* different localities. (B) Results of the top 30 genus‐level clustering heatmap of relative abundance between the gut microbiota of *Lycodon rufozonatus* and *Lycodon rosozonatus* from different localities.

We plotted the relative abundance of the top 10 phyla and the top 30 genera of the enteric flora of *L. rufozonatus* from the four areas and *L. rosozonatus* from the Hainan using a clustered heatmap. Five phyla were detected: Fusobacteriota, Cyanobacteriota, Bacteroidota, Campylobacterota, and Thermodesulfobacteriota (Figure 8A). At the genus level, thirteen genera, namely, *Pseudomonas*, *Flavobacterium*, *Bacteroides*, *Proteus*, *Alcaligenes*, *Morganella*, *Enterocloster*, *Klebsiella*, *Enterobacter*, *Citrobacter*, *Aeromonas*, *Clostridium*, and *Fusobacterium*, exhibited notably high or low bacterial populations (Figure 8B). As shown in the clustering heatmap, the relative abundance of colonies at the phylum and genus levels differed between the *L. rufozonatus* from the four sampling areas and the *L. rosozonatus* gut microbiota from the Hainan sampling area.

**FIGURE 9:**
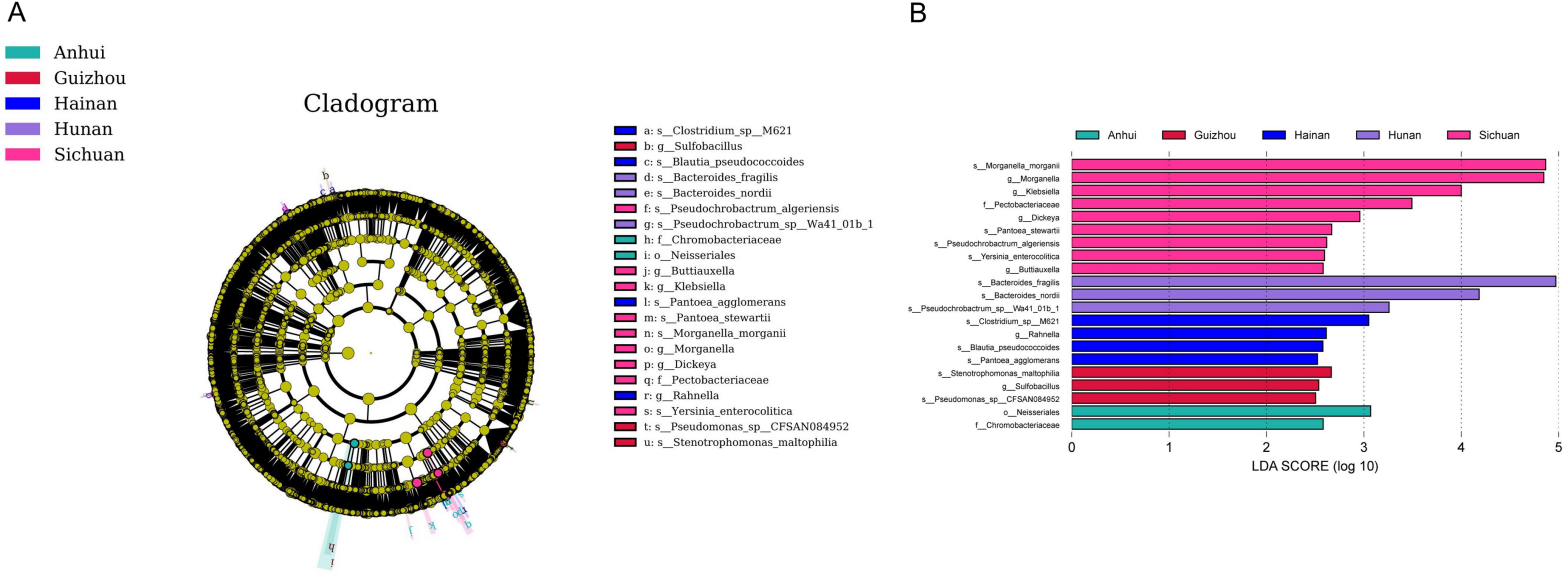
(A) LEfSe (linear discriminant analysis effect size) analysis was conducted to compare the composition of intestinal microbiota between *Lycodon rufozonatus* and *Lycodon rosozonatus* from different localities. The analysis included the clustering of intestinal microbiota and a class evolutionary tree. (B) Linear discriminant analysis (LDA > 2.5) scores of various bacteria at different taxonomic levels were examined.

Differential analyses by LEfSe (LDA effect size) showed that *L. rufozonatus* from Guizhou had 1 significant group of intestinal microbiota, *Sulfobacillus*; from Sichuan had 5 significant groups of gut microbiota, *Morganella*, Klebsiella, *Pectobacteriaceae*, *Dickeya*, and *Buttiauxella*; from Anhui, there were two significant groups of *enterobacteria*, *Neisseriales* and *Chromobacteriaceae*; and from Hainan, there was one important group of *enterobacteria* in the *enterobacterial* flora of *L. rosozonatus*, *Rahnella*. The gut microbiota of the sampling area in Hunan did not show a dominant flora and was therefore not analyzed further (Figure 9).

### 3.5 Carbohydrate CAZy Annotation Analysis

This study used the CAZy database to annotate the functions of protein sequences. CAZy database annotation revealed the presence of six CAZy functional gene categories in *L. rufozonatus* across four sampling areas and in *L. rosozonatus* in the Hainan sampling area. These categories included glycoside hydrolases (GHs), glycosyltransferases (GTs), carbohydrate esterases (CEs), polysaccharide cleavage enzymes (PLs), auxiliary module enzymes (AAs), and carbohydrate binding modules (CBMs). The annotation included a total of 336 CAZy gene family clusters, encompassing 131 GHs, 92 GTs, 52 CBMs, 30 PLs, 19 CEs, and 12 AAs family clusters.

**FIGURE 10:**
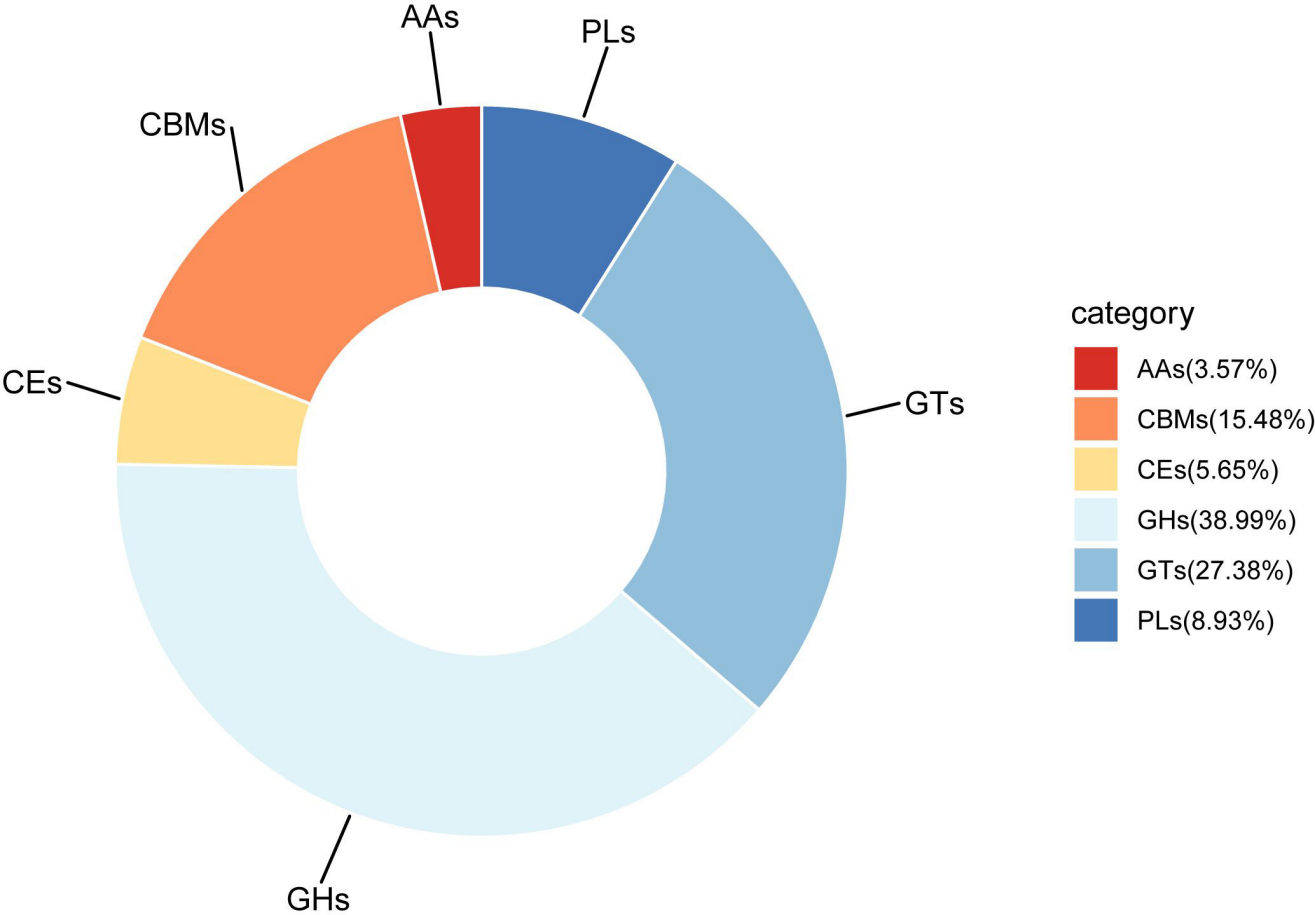
Pie chart of the abundance of six enzymes obtained by CAZy annotation of the gut microbiota of *Lycodon rufozonatus* from Guizhou, Sichuan, Hunan and Anhui, and *Lycodon rosozonatus* from Hainan. AAs, auxiliary activities; CBMs, carbohydrate‐binding modules; CEs, carbohydrate esterases; GHs, glycoside hydrolases; GTs, glycosyltransferases; PLs, polysaccharide lyases.

According to the CAZy annotation results (Figure 10), glycoside hydrolases (GHs) and glycosyltransferases (GTs) are the predominant functional proteins involved in carbohydrate metabolism, representing 45.72% and 37.31% of the total annotations, respectively. Additionally, carbohydrate‐binding protein modules (CBMs) and carbohydrate esterases (CEs) are relatively abundant, comprising 13.26%. These genes play a role in promoting carbohydrate metabolism. The abundance of auxiliary activities (AAs) and polysaccharide lyases (PLs), which influence polysaccharide catabolism, was low, at 3.7%. The annotation results indicated that the carbohydrate metabolism genes in the intestinal microbiota of *L. rufozonatus* from the four sampling areas and *L. rosozonatus* from the Hainan sampling area primarily participate in the synthesis of GHs and GTs. Additionally, some genes provide CBMs and assist in the degradation of PLs.

**FIGURE 11:**
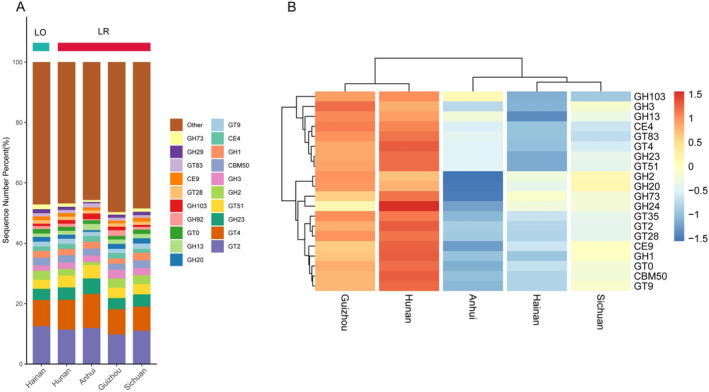
(A) Relative abundance stacking diagram of CAZy‐annotated gut microbiota of *Lycodon rufozonatus* from Guizhou, Sichuan, Hunan, and Anhui; and *Lycodon rosozonatus* from Hainan. (B) Statistical heatmap of the top 20 enzymes in abundance.

In *L. rufozonatus* from the four sampling areas and *L. rosozonatus* from the Hainan sampling area, the relative abundance of annotated CAZy functional genes showed similar proportions across sampling areas (Figure 11A); however, there was significant variability in the abundance of different enzyme categories within each sampling area (Figure 11B). In each sampling area, the top 20 CAZy functional genes belonged to four major enzyme families. *L. rufozonatus* from Guizhou and Hunan had the highest relative abundance of CAZy functional genes. The top five enzyme categories in all sampling areas included three glycosyltransferases (GT2, GT4, and GT51), which are responsible for specific sugar transfer reactions in biosynthesis, and two glycoside hydrolases (GH2 and GH23), which are mainly involved in the hydrolysis of glycosidic bonds to degrade carbohydrate compounds.

**FIGURE 12:**
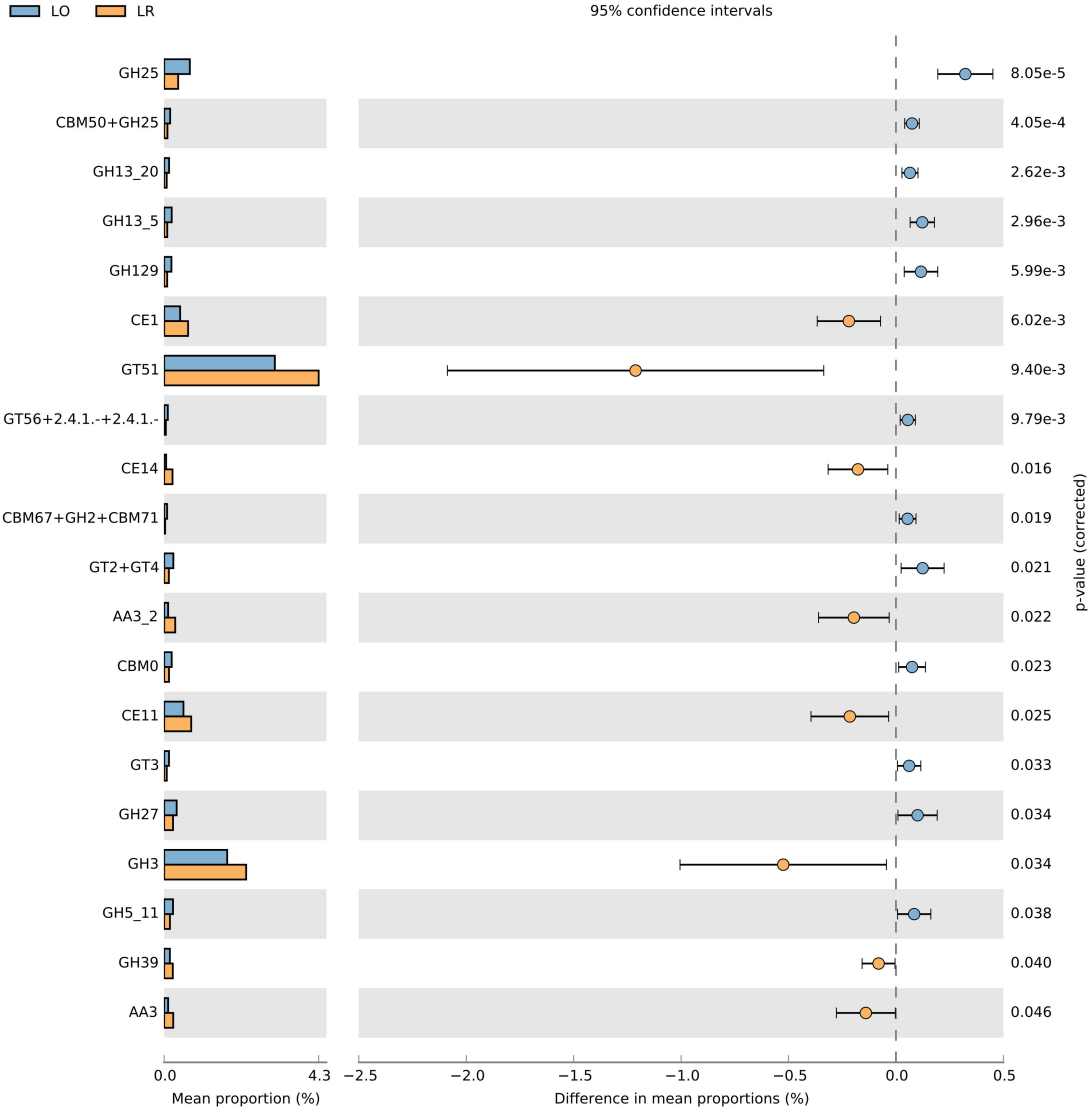
Comparison of carbohydrate enzyme families with significant differences between gut microorganisms of *Lycodon rufozonatus* and *Lycodon rosozonatus* from different localities.

Using a t test based on the database annotation results, we compared the differences in carbohydrate enzyme families between the gut microbiota of *L. rufozonatus* from the four sampling areas and that of *L. rosozonatus* from the Hainan area (Figure 12). The analysis revealed significant differences among 9 families of glycoside hydrolases (GHs), 4 families of carbohydrate‐binding modules (CBMs), 3 families of glycosyltransferases (GTs) and carbohydrate esterases (CEs), and 2 families of auxiliary activities (AAs). Among them, the top 20 most abundant enzyme families, GT51 and GH3, were significantly more abundant in *L. rufozonatus* than in *L. rosozonatus*. GH3 enzymes carry out a range of functions, including cellulosic biomass degradation, plant and bacterial cell wall remodeling, energy metabolism and pathogen defense. There are two classes of enzymes within the GT51 family: monofunctional (GT51 module only) and bifunctional (GT51 module linked to a penicillin‐binding transpeptidase module). These enzymes utilize the Und‐PP‐MurAc‐(GlcNAc)‐pentapeptide as a sugar donor.

## 4 Discussion

Increasing numbers of studies of gut microbiota in vertebrates have been reported in recent years, coinciding with the rapid development of high‐throughput sequencing (next‐generation sequencing) technologies (Huang et al. 2017; Levin et al. 2021; Tong et al. 2020; Waite and Taylor 2014). This novel sequencing method enhances our understanding of the composition and structure of intestinal microorganisms in animals, enabling us to monitor animal health more effectively and develop informed conservation strategies. The diversity of gut microorganisms can promote the overall health and nutritional metabolism of the host and regulate immune function and reproductive behavior (Phillips et al. 2012; Wei, Wang, and Wu 2015). However, our understanding of reptilian gut microbiomes is still relatively poor compared to birds or mammals (Delport et al. 2016; Lee et al. 2020; Nelson et al. 2013; Sun et al. 2019). Moreover, most studies on reptile gut microbiota focus on captive populations, with limited research on wild reptiles in their natural environments (Costello et al. 2010). In this study, we collected gut microbiota samples of *L. rufozonatus* from four different areas including Guizhou, Sichuan, Hunan and Anhui, as well as from *L. rosozonatus* from Hainan. The results indicated that there were no significant differences in the composition or diversity of the gut microbiota between the two species.

Many studies have identified significant differences in the primary gut microbiota among various snake species. For example, the intestinal flora of Viperidae and Colubridae primarily consists of three bacterial phyla: Bacteroidetes, Proteobacteria, and Firmicute (Colston, Noonan, and Jackson 2015; Hu, Yang, and Tian 2024; McLaughlin, Cochran, and Dowd 2015; Wei et al. 2022). Although the composition of their intestinal flora is similar, the dominant bacterial phyla in their intestinal flora are different between different snake species. Such as, in *Crotalus horridus*, the dominant bacterial phyla are Proteobacteria and Firmicutes, while in *Ptyas mucosus* and *Elaphe carinata*, they are Bacteroidetes and Proteobacteria (Zhang et al. 2021; Huttenhower et al. 2012). In the study of the intestinal flora of pythons, it was found that the dominant bacterial phyla of their intestinal flora were also Bacteroidetes and Firmicutes. This shows gut microbial populations exhibit significant variability across numerous snake species, including closely related ones (Hoffbeck et al. 2023). However, this conclusion contradicts the findings of our experiment. Here we found that the dominant gut microbiota of *L. rufozonatus* from Guizhou, Sichuan, Hunan, and Anhui, as well as *L. rosozonatus* from Hainan, were from the phyla Pseudomonadota, Bacteroidota, and Bacillota. Therefore, we speculated that the composition and diversity of the gut microbiota of *L. rufozonatus* and *L. rosozonatus* did not change much due to their similar feeding habits.

Since interspecies differences in intestinal microbiota are influenced by multiple factors, such as diet, habitat, and genetics, it is important to consider these variables when interpreting the results (Krishnankutty et al. 2018; Qin et al. 2019; Zhang et al. 2019). For instance, a study examining the gut microbiota of 91 distinct reptile species revealed that the primary factor influencing the microbiota of reptiles was the host diet, with host identity, conservation status, environmental factors, and captivity status also playing significant roles. Research suggests that the introduction of novel food sources is a primary driver of species diversification and concurrent evolution of the gut microbiota (Ley, Lozupone et al. 2008). Additionally, dietary changes during ontogeny are expected to induce alterations in the gut microbiota composition. This phenomenon extends to lizards as well. In a study on *Diploderma splendidum*, it was showed that diet significantly influenced the bacterial community (Tian et al. 2020). The aforementioned studies collectively demonstrated that the type of diet plays an important role in determining the composition of the gut microbiome (Tang et al. 2019).

Additionally, a separate study on reptiles investigated the gut microbiota of three distinct sea turtle species (*Caretta caretta*, *Lepidochelys kempii*, and *Chelonia mydas*), revealing variations in microbiota composition among species and across different gut regions (Forbes et al. 2023). Although the fecal diversity differed, the three most abundant bacterial groups in the feces consisted of Proteobacteria, Bacteroidetes, and Firmicutes. These findings suggest that, although the composition of the gut microbiota may appear similar between the two species, significant differences exist in the diversity of bacterial flora. In contrast, despite the vast geographical distance between the habitats of the two species of snakes in this study, they share a consistent core gut microbiota, with no significant differences in the diversity of their gut microbiota. This finding encourages further exploration of the biological functions carried out by the gut microbiota.

In this study, the phylum Pseudomonadota was the most abundant bacterial group in all samples from the five sampling areas. Pseudomonadota, a facultative anaerobic bacterium, primarily participates in fat uptake, maintains intestinal anaerobic environment homeostasis, decomposes various organic compounds, and facilitates host nutrient absorption (Moon et al. 2018). In a healthy gut, Pseudomonadota combats infections or inflammation, playing a protective role in the immune response (Shin, Whon, and Bae 2015). Pseudomonadota constitutes 48.93% to 65.52% of the gut microbiota in *L. rufozonatus* from Guizhou, Sichuan, Hunan and Anhui and *L. rosozonatus* from Hainan and similarly dominates the gut microbiota in other reptiles, such as these four species of reptiles (*Amblyrhynchus cristatus*, *Liolaemus parvus*, *Liolaemus ruibali*, and *Phymaturus wilningsi*). The abundance of Proteobacteria in the intestine varies from 19.1% to 56.4% (Kohl et al. 2017; Costello et al. 2010; He et al. 2018; Xiao et al. 2021). The gut microbiota of *L. rufozonatus* and *L. rosozonatus* is dominated by Pseudomonadota, whereas in mammals, Bacteroidetes and Firmicutes are dominant (Ley, Lozupone et al. 2008). In a recent study, the genera Pseudomonas and Firmicutes were found to dominate the bacterial communities of insects and birds (Hird et al. 2014), suggesting that phylogenetic relationships may influence the composition of the gut microbiota. *Citrobacter*, a genus in Pseudomonadota, accounts for 8.06%–11.18% of the gut microbiota of *L. rufozonatus* and *L. rosozonatus*. *Citrobacter* comprises facultative anaerobes known for their animal pathogenicity and potential to cause enteritis (Mundy et al. 2005); its abundance is correlated with the host's carnivorous diet (Stiles and Ng 1981). For instance, the carnivorous bird Falco tinnunculus harbors a significant population of Enterobacteriaceae bacteria in its intestine (Zhang et al. 2022). Based on this, some studies suggest that the gut microbiota of snakes are more similar to those of birds than mammals, indicating that phylogenetic relationships may influence the composition of gut microbiota.

Among the five sampling areas, the relative abundance of Bacillus and Bacteroidetes ranked second and third, respectively. Previous study indicated that the ratio of Bacillota to Bacteroidota in two species of *Elaphe* (*Elaphe carinata* and *Elaphe taeniura*) correlated with energy consumption, body weight changes, and various influencing factors, higher ratios in host animals facilitate rapid energy acquisition (Qu et al. 2022). In the intestines of *L. rufozonatus* and *L. rosozonatus*, Bacteroidota and Bacillota constituted 8.94%–23.27%, respectively. The resulting ratio is approximately 0.3, which contrasts with the ratio observed in the aforementioned study. Nevertheless, two species of *Elaphe* are large snakes with abundant food sources, necessitating increased energy intake to support their growth and development. Bacteroidota ranked second in the gut microbiota of *L. rufozonatus* and *L. rosozonatus* (21.67 ± 14.34%). This phylum encompasses strict anaerobic bacteria capable of polysaccharide decomposition, thereby enhancing nutrient utilization, digestion, and complex carbohydrate utilization (Spence, Wells, and Smith 2006). Additionally, it likely aids in the development of the host's intestinal mucosa and immune system while maintaining intestinal microecological balance (Sears 2005). Bacillota bacteria are prevalent in the intestinal microbiota of vertebrates and insects and contribute significantly to host digestion and energy metabolism (Ley, Lozupone et al. 2008).

Comparison of the intestinal microbial functions between *L. rufozonatus* and *L. rosozonatus* revealed their predominant involvement in glycosyl synthesis, glycosyl modification, and signal transduction, regulating carbohydrate synthesis and decomposition alongside controlling various physiological processes such as cell metabolism, growth, and differentiation. Although there are differences in their abundance during carbohydrate metabolism, the composition of the enzyme families involved remains consistent. We hypothesize that, in response to various survival challenges, additional enzyme families may be required to ensure complete carbohydrate synthesis and breakdown.

## 5 Conclusions

This study used high‐throughput sequencing technology and 16S rRNA sequence analysis technology to explore the gut microbiota of two closely related snake species *L. rufozonatus* and *L. rosozonatus*. The results showed there was no significant differences in the composition or bacterial diversity of the gut microbiota between the two closely related snake species. The dominant bacterial phyla of *L. rufozonatus* and *L. rosozonatus* at the phylum level included Pseudomonadota (gamma‐pseudomonadota of the γ subgroup of Proteobacteria), Bacteroidota and Bacillota. However, there were five other bacterial phyla: Fusobacteriota, Cyanobacteriota, Bacteroidota, Campylobacterota and Thermodesulfobacteriota. At the genus level, there were 13 genera, including *Pseudomonas*, *Flavobacterium*, *Bacteroides*, *Proteus*, *Alcaligenes*, *Morganella*, *Enterocloster*, and *Klebsiella*. There were differences in the relative abundances of *Enterobacter*, *Citrobacter*, *Aeromonas*, *Clostridium*, and *Fusobacterium*. In the subsequent annotation of carbohydrates, the enzyme clusters had the same composition but differed in abundance. It is speculated when *L. rufozonatus* and *L. rosozonatus* face different survival choices, more enzyme families may be needed to complete the synthesis and decomposition of carbohydrates. Overall, our results provide a basis for understanding the coevolutionary relationships between gut microbes and hosts of two closely related species and will provide insights into the evolution and ecology of snakes and the development of conservation measures.

## Author Contributions

**Fei Zhu:** conceptualization (equal), funding acquisition (lead), investigation (equal), methodology (equal), resources (equal), writing – review and editing (lead). **Ke Sun:** conceptualization (equal), data curation (equal), investigation (equal), methodology (equal), writing – original draft (lead). **He Zhang:** conceptualization (equal), investigation (equal). **Jing Lu:** conceptualization (equal), writing – review and editing (equal). **Peng Guo:** conceptualization (equal), resources (equal), writing – review and editing (equal). **Jiaqi Zhang:** conceptualization (equal), data curation (equal). **Yu Xu:** conceptualization (equal), writing – review and editing (equal). **Bing Lyu:** conceptualization (equal), writing – review and editing (equal).

## Ethics Statement

All samples were collected following Chinese regulations for the Implementation of the Protection of Terrestrial Wild Animals (State Council Decree [1992] No. 13). All the experiments and methods were performed in accordance with the Animal Research: Reporting of In Vivo Experiments (ARRIVE) guidelines. All the methods were carried out in accordance with relevant guidelines and regulations. Animal handling and experimental procedures were approved by the Ethics Committee of Guizhou Normal University (Permission number: 2024030001).

## Conflicts of Interest

The authors declare no conflicts of interest.

## Supporting information


**Table S1.** Basic physiological information of samples.
**Table S2.** Alpha diversity index of each sample.

## Data Availability

All data are available in the NCBI, and the link to the data is https://www.ncbi.nlm.nih.gov/bioproject/PRJNA1108955 (A series of SRA accession: SRR29213544‐SRR29213564).
