# Supplementary material for: Comparative Analyses of Lycodon rufozonatus and Lycodon rosozonatus Gut Microbiota in Different Regions
Source: Ecol Evol. 2024 Oct 22;14(10):e70480. doi: 10.1002/ece3.70480 (PMC11495892; doi:10.1002/ece3.70480)
Supplement: Supplementary file 1 — Table S1. Basic physiological information of samples. Table S2. Alpha diversity index of each sample. [file ECE3-14-e70480-s001.docx]

**Table S1:** Basic physiological information of samples

| Location | Sample | Female/Male | Adult/Juvenile | Health/Sickness |
| --- | --- | --- | --- | --- |
| hainan | EMS0676 | male | Adult | Health |
| hainan | EMS0689 | male | Adult | Health |
| hainan | EMS0690 | male | Adult | Health |
| guizhou | EMS0691 | male | Adult | Health |
| guizhou | EMS0692 | male | Adult | Health |
| guizhou | GP10137 | Female | Adult | Health |
| guizhou | GP10138 | Female | Adult | Health |
| guizhou | GP9686 | male | Adult | Health |
| hunan | GP10142 | male | Adult | Health |
| hunan | GP10143 | Female | Adult | Health |
| hunan | GP10144 | male | Adult | Health |
| hunan | GP10161 | Female | Adult | Health |
| hunan | GP10829 | Female | Adult | Health |
| sichuan | GP10817 | Female | Adult | Health |
| sichuan | GP10819 | Female | Adult | Health |
| sichuan | GP10835 | Female | Adult | Health |
| anhui | GP12533 | male | Adult | Health |
| anhui | GP12570 | Female | Adult | Health |
| anhui | GP12571 | Female | Adult | Health |
| anhui | GP9292 | male | Adult | Health |
| anhui | EM062406 | male | Adult | Health |

**Table S2:** Alpha diversity index of each sample

| sample | shannon | simpson | chao1 | ACE | observed_species | goods_Coverage |
| --- | --- | --- | --- | --- | --- | --- |
| EM062406 | 5.479001 | 0.962927 | 11666.84 | 11534.56 | 11364 | 0.9996692 |
| EMS0676 | 4.833666 | 0.958634 | 11415.84 | 11294.38 | 10904 | 0.9994128 |
| EMS0689 | 3.473269 | 0.938314 | 9175.087 | 9164.102 | 7295 | 0.9986903 |
| EMS0690 | 3.488985 | 0.920928 | 9521.893 | 9481.608 | 7818 | 0.9987444 |
| EMS0691 | 5.030447 | 0.957634 | 11633.93 | 11483.67 | 11192 | 0.9995136 |
| EMS0692 | 3.938161 | 0.950903 | 9239.973 | 9171.42 | 7562 | 0.9988029 |
| GP9292 | 3.740728 | 0.916633 | 9994.531 | 9789.287 | 8655 | 0.9989818 |
| GP9686 | 4.836486 | 0.962121 | 11045.68 | 10944.09 | 10409 | 0.9992763 |
| GP10137 | 3.956472 | 0.942569 | 9925.885 | 9855.477 | 8517 | 0.9988416 |
| GP10138 | 3.962632 | 0.938426 | 10313.72 | 10208.47 | 8686 | 0.9987549 |
| GP10142 | 3.421468 | 0.935947 | 8476.138 | 8785.654 | 5899 | 0.9985145 |
| GP10143 | 3.546732 | 0.933353 | 10267.95 | 10366.24 | 8491 | 0.9985692 |
| GP10144 | 3.896025 | 0.943896 | 10729.55 | 10675.86 | 9378 | 0.9987807 |
| GP10161 | 3.844911 | 0.944093 | 10170.96 | 10233.1 | 8383 | 0.9985809 |
| GP10817 | 3.32775 | 0.922389 | 9527.348 | 9811.643 | 7332 | 0.9984714 |
| GP10819 | 4.045607 | 0.945231 | 9731.431 | 9673.263 | 7888 | 0.9987469 |
| GP10829 | 3.981164 | 0.947892 | 10421.59 | 10408.05 | 9128 | 0.9988084 |
| GP10835 | 4.387701 | 0.956386 | 10341.32 | 10142.77 | 9203 | 0.9990396 |
| GP12533 | 4.515728 | 0.950181 | 11329.24 | 11223.52 | 10540 | 0.9991921 |
| GP12570 | 3.404304 | 0.925487 | 9616.449 | 9945.816 | 7472 | 0.9984616 |
| GP12571 | 3.990146 | 0.934281 | 10908.56 | 10900.9 | 9896 | 0.9989424 |
